# Enhanced optoelectronic quality of perovskite thin films with hypophosphorous acid for planar heterojunction solar cells

**DOI:** 10.1038/ncomms10030

**Published:** 2015-11-30

**Authors:** Wei Zhang, Sandeep Pathak, Nobuya Sakai, Thomas Stergiopoulos, Pabitra K. Nayak, Nakita K. Noel, Amir A. Haghighirad, Victor M. Burlakov, Dane W. deQuilettes, Aditya Sadhanala, Wenzhe Li, Liduo Wang, David S. Ginger, Richard H. Friend, Henry J. Snaith

**Affiliations:** 1 Clarendon Laboratory, Department of Physics, University of Oxford, Parks road, Oxford OX1 3PU, UK; 2 Mathematical Institute, University of Oxford, Woodstock Road, Oxford OX2 6GG, UK; 3 Department of Chemistry, University of Washington, Box 351700, Seattle, Washington 98195-1700, USA; 4 Cavendish Laboratory, Department of Physics, University of Cambridge, 19 JJ Thomson Avenue, Cambridge CB3 0HE, UK; 5 Department of Chemistry, Tsinghua University, Beijing 100084, China

## Abstract

Solution-processed metal halide perovskite semiconductors, such as CH_3_NH_3_PbI_3_, have exhibited remarkable performance in solar cells, despite having non-negligible density of defect states. A likely candidate is halide vacancies within the perovskite crystals, or the presence of metallic lead, both generated due to the imbalanced I/Pb stoichiometry which could evolve during crystallization. Herein, we show that the addition of hypophosphorous acid (HPA) in the precursor solution can significantly improve the film quality, both electronically and topologically, and enhance the photoluminescence intensity, which leads to more efficient and reproducible photovoltaic devices. We demonstrate that the HPA can reduce the oxidized I_2_ back into I^−^, and our results indicate that this facilitates an improved stoichiometry in the perovskite crystal and a reduced density of metallic lead.

## Abstract (web summary)

An imbalance in I/Pb stoichiometry is thought to lead to defects in metal halide films. Here, Zhang *et al*. show that the addition of hypophosphorous acid in the precursor solution can significantly improve the film quality and enhance the photoluminescence intensity, leading to improved photovoltaic devices.

## Graphical abstract

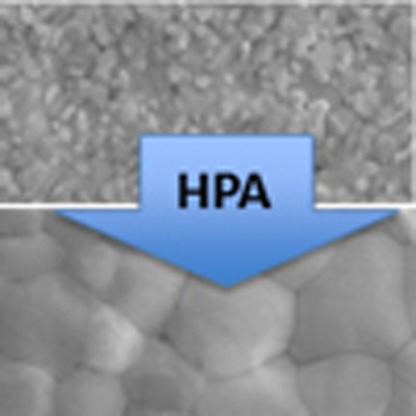

Metal halide perovskite semiconductors, such as CH_3_NH_3_PbI_3_ and mixed halide variants, have recently become a commercially relevant material for photovoltaic (PV) and light emission applications^1^,^2^,^3^,^4^. With photovoltaic solar to electrical power conversion efficiencies (PCE) surpassing 20% on laboratory-type cells^5^, they are already promising to emerge as a close competitor to crystalline silicon solar cells. Easy fabrication of polycrystalline perovskite thin films with high crystallinity has been central to the rapid advancement of the solar cell performance. However, there still remains much scope for further improvement since there exists significant heterogeneity in the polycrystalline films^6^, and little is known about the defect chemistry^7^,^8^,^9^,^10^. Impurities and defects introduced during the fabrication process could potentially act as non-radiative recombination centres and subsequently harm the photophysical properties of the perovskite films. High-performance semiconductor devices generally require high purity and low-defect materials to deliver the best performance^11^. Passivation of electronic defects at grain boundaries has been central to the advancement of thin-film solar technologies based on copper indium gallium selenide (CIGS)^12^ and CdTe^13^, in addition to polycrystalline silicon^14^. The energetic potential barrier at perovskite grains has been shown to be low, in the region of thermal energy^15^. However, recent reports of large crystalline domains delivering enhanced charge-carrier lifetimes point towards the detriment of grain boundaries and grain surfaces are additionally likely to be the source of mobile ionic species responsible for hysteresis in the solar cells current-voltage curves^16^,^17^,^18^. More recently still, deQuilettes *et al*. have examined polycrystalline perovskite thin films and observed fast photoluminescence (PL) quenching at grain boundaries, which is attributed to a large densities of non-radiative centres in these regions^6^. If we compare polycrystalline thin films to single crystals of MAPbI_3_ (ref. 18), then single crystals grown very slowly from solution have been shown to have defect densities of as low as 10^9^ to 10^10^ cm^−3^. In comparison, polycrystalline thin films general have defect densities in the order of 10^16^ cm^−3^ indicating much room for improvement^19^. We do note that provided the defect density is lower than the density of free carriers generated under photovoltaic operation under sunlight, their impact on the electronic operation will be minimal. However, this is likely to require reducing the density to the order of 10^14^ or lower in the polycrystalline thin films. Hence understanding what parameters in the film preparation influence defect generation, and what means can be devised to reduce defect density will be central to advancing the performance and large-area reproducibility of perovskite solar cells.

To date, several techniques have been developed for making perovskite thin films including one-step solution coating^2^,^3^,^20^, sequential deposition^21^, vapour phase deposition^22^ or any combination thereof^23^. Among these, one-step solution coating is relatively advantageous, since it relies on inexpensive deposition equipment and is versatile on controlling the deposition conditions and the composition of perovskite materials^12^,^13^. However, fabrication of the perovskite thin films is greatly influenced by the composition of precursor solution since the excess organic compound or the by-products generated during the perovskite crystallization may directly or indirectly influence the crystallization kinetics and also the as-crystallized film morphology^24^. Several techniques have been established to improve the perovskite thin-film morphology by introducing additives in the precursor solution, such as methylammounium chloride (MACl)^25^ and 1,8-diiodooctane (DIO)^26^. The role of these additives has been mainly attributed to tuning the crystallization kinetics, whilst their effect on the stoichiometric composition and defect density of perovskite films remains unclear. Our recent work also demonstrated that the crystallization kinetics could be easily tuned by replacing conventional lead halide (lead iodide or lead chloride)-based precursor with lead acetate^27^, and ‘ultrasmooth' and pin-hole free perovskite thin films are easily obtained without use of any further additives or post treatment, leading to improved device performance in the planar heterojunction architecture.

In addition to the perovskite thin-film morphology, recent reports have shown that the perovskite solar cell performance is very sensitive to the stoichiometry of organic halide/lead salt, due to the presence of colloids, rather than a true solution, in the starting salt/solvent mixture^28^. This latter observation adds a further level of complexity to the single-step solution coating method for producing perovskite thin films, since the nature of the final film is not only sensitive to the casting and curing conditions, but may already be largely predetermined by the nature of the colloids in the precursor solution. It is also well known that organic iodides such as methylammonium iodide (MAI), are hygroscopic, not very stable in air and under light irradiation, especially after long-term storage, with the instability attributed to the oxidation of I^−^ into I_2_ (ref. 29). When it is used to prepare perovskite precursor solution, the accurate stoichiometric ratio of organic halide to lead salt may not be achieved, which will potentially introduce defects in perovskite material and can be detrimental to device performance. In addition, if there exist polyiodide complexes ([PbI_4_]^2−^, [PbI_5_]^3−^ or [PbI_6_]^4−^) and/or colloids in the starting solution^28^, then the precise complex and colloid thereof may be extremely sensitive to the stoichiometry of the I/Pb in solution and we could envisage that halide vacancies or other defects induced by non-stoichiometry could be ‘locked in' to the material, even from solution. Furthermore, during the crystallization on heating, oxidization of I^−^ may occur, which could result in the generation of bulk defects within the crystal. The best way to address this issue would be to use extremely pure MAI by avoiding any light, heat and moisture exposure. In addition I^−^ ions in aprotic solvent, under oxidizing conditions, may form I_2_ in the precursor solution, introducing another source of violation in I/Pb stoichiometry^30^,^31^,^32^.

Taking note of the above considerations, here we show the addition of a strong reducing agent, hypophosphorous acid (HPA), which is commonly added as a stabilizer in commercial hydroiodic acid (HI) solution^33^ into the precursor solution in an attempt to retard such oxidation of iodide. We demonstrate that the resultant CH_3_NH_3_PbI_3_ perovskite films have greatly enhanced emissive properties by reducing the non-radiative recombination centres. This additive however does not appear to be present in the perovskite crystal after the crystallization process, but simply assists in the crystallization of high quality perovskite thin films, leading to a substantial increase in the PL quantum efficiency (PLQE) and subsequently greatly improved solar cell performance and reproducibility.

## Results

### Morphological and crystallographic characterization

In this work we employed the lead acetate trihydrate (PbAc_2_·3H_2_O) based precursor route, which is processed from a 3:1 molar ratio of MAI:PbAc_2_·3H_2_O, as we have previously described^27^. In contrast to our previous work^27^ where we employed lab-made MAI, here we employed commercially available MAI to ensure a high level of purity in the starting material and easier reproducibility for others. Concerning the lab-made MAI, we have noticed that significant differences in perovskite film formation and crystallization exist depending upon which batch of MAI is employed, and precisely how the MAI has been synthesized and purified. For our previous ‘standard' lab-made MAI we found best solar cell results were obtained if the MAI was dried, but not purified via recrystallization following the synthesis. This indicates that an ‘impurity' may be present in the as-synthesized MAI which could have a beneficial influence upon the crystallization and optoelectronic properties of the perovskite. As a means to investigate the impurities present in the MAI we performed X-ray photoemission spectroscopy (XPS) measurements on recrystallized and as-synthesized MAI. We show the XPS spectra in the supplementary Fig. 1, where we can observe a trace signal of phosphorous. MAI is synthesized by mixing MA with HI, and a stabilizer present in the HI is the reducing agent, HPA. Hence HPA is a likely culprit for the phosphorous signal.

We therefore investigate whether adding the reducing agent, HPA, to the perovskite precursor solution can have a beneficial influence on the as deposited perovskite films when employing purified MAI. Below we perform a morphological and crystallographic characterization of the perovskite thin films obtained from the perovskite precursor solution with and without HPA (control).

**Figure 1:**
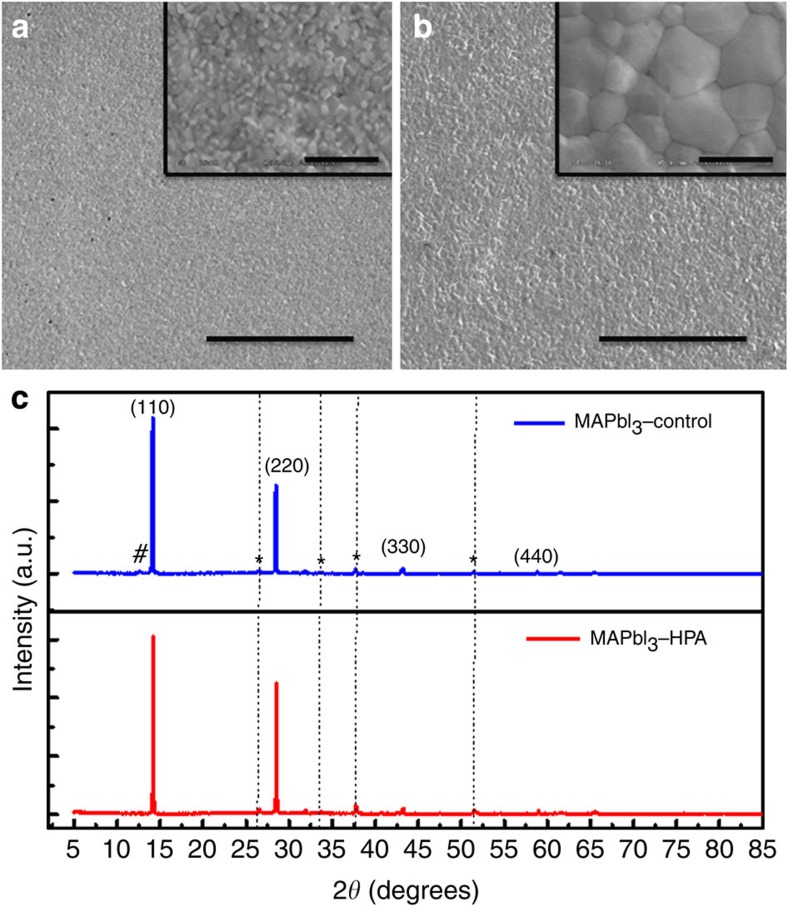
Morphological and crystallographic analysis. SEM images for the perovskite thin films deposited on compact TiO_2_ (c-TiO_2_) coated FTO glass prepared from the precursor solution without (**a**) and with (**b**) HPA, with scale bars of 20 μm in the images and 1 μm in the insets, and corresponding XRD spectra (**c**). Note that the scattering peak from PbI_2_ impurity, denoted #, is absent from the material processed with HPA.

In Fig. 1a,b we show the SEM images of the perovskite thin films. We observe that both samples show full coverage on the substrate with the absence of pin-holes. From the high-magnification images which we show in the insets, the perovskite grain size appears to be significantly enlarged by the addition of HPA, increasing from an average value of 168 to 769 nm for control and HPA-modified samples, respectively. We note that the SEM image is only sensitive to the surface of the film. We presume that the grains are predominantly propagating throughout the thickness of the film, but cannot exclude the possible presence of smaller crystallites at the buried interface.

From the XRD spectra, which we show in Fig. 1c, it is evident that the materials fabricated from perovskite solutions crystallize in a tetragonal crystal structure. X-ray diffraction reflection confirms the CH_3_NH_3_PbI_3_ perovskite structure with lattice parameter with *a*=*b*=8.8724 Å and *c*=12.5475 Å for the control sample and *a*=*b*=8.8650 Å and *c*=12.5370 Å for the sample with the HPA additive. An important observation we make from the X-ray diffraction spectra is that the PbI_2_ impurity, characteristic by the scattering peak, denoted #, at around 2*θ*=12.6°, is present in the control sample but appears to be absent in the sample processed with HPA.

### Photophysical properties of the perovskite thin films

**Figure 2:**
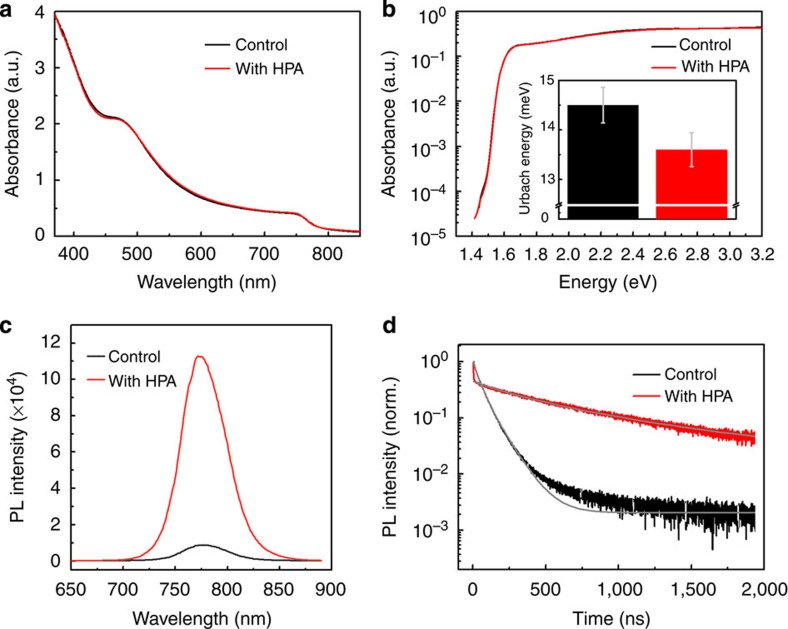
Photophysical properties of the perovskite films. (**a**) UV–vis absorption spectra of ∼200-nm-thick perovskite thin films coated on glass substrates prepared from the precursor solution without (control) and with HPA. (**b**) PDS spectra of the perovskite film without and with HPA deposited on quartz substrate. The inset shows the average Urbach energies for these samples with s.d. (**c**) Steady-state and (**d**) time-resolved photoluminescence (PL) spectra for the perovskite thin films deposited on glass prepared from the precursor solution without (control) and with HPA. Grey lines are the model fits (Supplementary Note 1).

We subsequently investigate the photophysical properties of the as-crystallized perovskite films. We do not discern any difference in the ultraviolet–visible spectra of the perovskite thin films with or without HPA, as we show in Fig. 2a.

The ultraviolet–visible absorption is not sensitive enough to measure the absorption in the sub-bandgap region. However, significant information related to the quality and defects in the semiconductor can be obtained by studying the sub-bandgap absorption spectra. For such measurements we employed photothermal deflection spectroscopy (PDS), which is a sensitive absorption measurement technique capable of measuring absorbance down to 10^−5^. Further, by fitting the exponential drop in absorption at the band edge we can extract the energetic disorder parameter, known as Urbach energy ‘*E*_*u*_', which is given by *A*=*A*_*0*_ exp(*E*/*E*_*u*_) where, *A* is the Absorbance, *A*_*0*_ is a constant for data fitting and *E* is the excitation energy^34^,^35^,^36^. Although to a non-expert the PDS spectra look very similar for the films processed with and without HPA, the films processed with HPA exhibit a sharper band-edge absorption rise compared to the control perovskite film (Supplementary Fig. 2a). The energetic disorder estimated using the Urbach energy fits to the band edge (Supplementary Fig. 2b,c) reveal a lower Urbach energy for the perovskite with HPA additive (∼13.5±0.3 meV) compared with the control perovskite film (14.5±0.4 meV) as we shown in the inset of Fig. 2b. This 1-meV difference is significant and indicates a higher level of electronic order in the HPA-processed films, and to the best of our knowledge Urbach energy of 13.5 meV is the lowest yet reported for lead halide perovskites^27^,^35^,^36^.

In Fig. 2c we show the steady-state PL spectra for the perovskite films and observe that the PL intensity of the perovskite film processed with HPA is an order of magnitude higher than that of the control perovskite film. We compare the PLQE of the respective perovskite films and find that the HPA-modified perovskite film shows an improvement in its PLQE reaching 11.6±0.7% as compared with 0.84±0.15% in the control film, following the similar trend as steady-state PL spectra.

These measurements collectively indicate that the electronic quality of the perovskite film processed with HPA is enhanced in terms of reduced energetic disorder and enhanced charge-carrier lifetime. To quantify this, we measure the time-resolved PL decay for both films and show the results in Fig. 2d. Fitting the PL decay dynamics to our model for charge recombination within perovskite films (see supplementary Note 1 for the details of data fitting), as described in detail by Stranks *et al*.^37^, we estimate a reduction in the filled trap density from 1.58 × 10^16^ to 3.25 × 10^15^ cm^−3^ for the control to the HPA-processed film. This is consistent with a reduction in the total trap density, which is indicative of fewer non-radiative decay pathways likely to be resultant from reduced density of defect sites when we process the films with HPA.

It is evident thus far that when HPA is introduced into the perovskite precursor solution, it produces films with much lower energetic disorder as compared with the control sample. However, it is still not clear as to how this is occurring. This reduction in defect density could either be due to the enlarged perovskite grain domains and hence a reduction of the defect density by simply reducing the grain boundary density, or by reducing the density of non-radiative recombination sites, which could be present within the bulk or at the surface of each grain, or at the grain boundaries.

**Figure 3:**
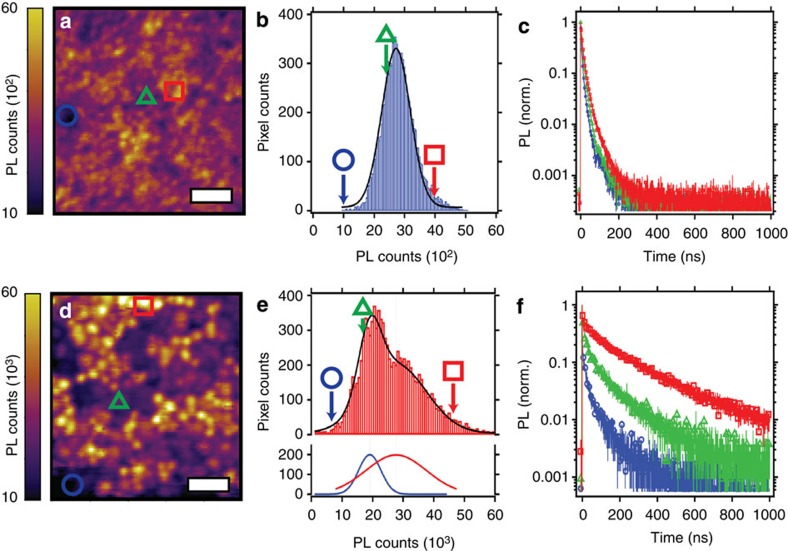
Fluorescence microscopy analysis. (**a**) A 10 × 10 μm fluorescence image (FI) of a control perovskite film on glass and (**d**) a perovskite film formed with HPA additive. (**a**,**d**) Scale bar, 2 μm. (**b**) Image histogram of FI in (**a**) fitted to a Gaussian function (black trace) and ((**e**), top) histogram of FI in (**d**) fitted to a sum (black trace) of two Gaussian functions (blue and red traces, (**e**) bottom). (**c**,**f**) Time-resolved PL decay curves of bright (red square), medium (green triangle), and dark (blue circle) PL intensity regions after excitation at 470 nm, 500 kHz, *ϕ*=1 μJ cm^−2^.

From the SEM images, we know that the grains do enlarge when the films are processed with HPA, but to further elucidate the microstructural changes controlling the photophysical properties, we use fluorescence microscopy to spatially resolve the PL intensity and PL decay dynamics. In Fig. 3a we show a 10 × 10 μm confocal fluorescence image of a control film. In the image histogram (Fig. 3b) we observe a fairly narrow distribution of uniformly dark domains (average PL counts ∼2,800). We measured the local time-resolved PL decay curves of three different regions (red square, green triangle and blue circle in Fig. 3a,b) of the film and observe fast PL dynamics across these separate regions (Fig. 3c). We also collected a fluorescence image of a 10 × 10 μm area of a film processed with HPA (Fig. 3d) under the same excitation fluence and in contrast, we observe an order of magnitude higher average PL intensity (average PL counts ∼26,000), a larger distribution of PL intensities (Fig. 3e), and slower recombination kinetics (Fig. 3f). Interestingly, we observe two distinct sets of domains in Fig. 3e. As we show clearly in the histogram in Fig. 3f, grains falling under the dark tail of the distribution (blue circle, Fig. 3d,e) have the fastest PL decay, grains close to the centre of the distribution (green triangle, Fig. 3d,e) have a slightly slower PL decay, which explains the fast component observed in the macroscopic PL, and the bright regions (red square) have the slowest decay. These results are consistent with observations made in high-performing CH_3_NH_3_PbI_3_(Cl) polycrystalline films^6^. Importantly, the trends we report here match the macroscopic time-resolved PL decays, which we presented in Fig. 2d. The slightly faster microscopic PL decays compared with the macroscopic PL decays can be attributed to the differences in excitation fluences. We interpret these measurements as indicative of non-radiative recombination sites within the perovskite films being reduced by processing with HPA, but cannot identify if this preferentially reduces sites within the grains, at their surfaces or boundaries. We note that there exists significant heterogeneity in the PL from the HPA-processed films, indicating that the HPA is not necessarily a remedy for this specific issue, or that our processing routine here has not enabled the HPA to homogeneously enhance the perovskite film, clearly there remains much scope for further improvement.

### Analysis of electronic trap states

Our recent study on correlating PL maps with electron microscope images has demonstrated that PL is quenched mostly at the surface and grain boundaries. This is consistent with defects responsible for non-radiative recombination predominately existing at the surfaces and grain boundaries^6^. To bring more clarity on the nature of the non-radiative recombination sites here, we performed kelvin probe based measurements to acquire surface photovoltage of both control and HPA-processed perovskite films coated on fluorine-doped tin oxide (FTO) glass.

**Figure 4:**
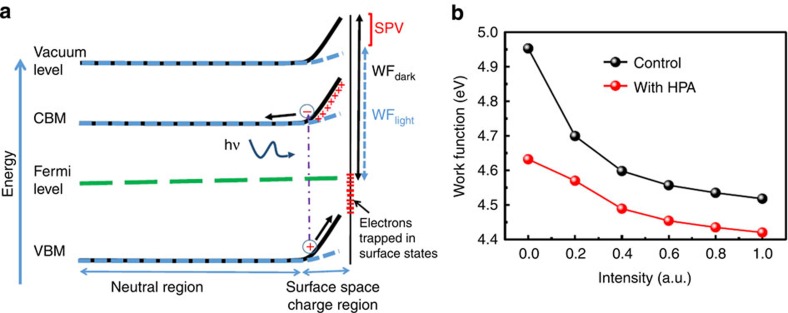
Kelvin probe measurements. (**a**) Schematic band diagram for the generation of surface photovoltage (SPV) at the perovskite surface (CBM, conduction band minimum, VBM, valence band maximum). The solid lines and the dotted lines represent the condition in the dark and light, respectively. (**b**) Surface work function of perovskite films on FTO as a function of light intensity; control perovskite film shows a decrease in work function by 430 mV, whereas the HPA-modified sample shows a decrease of 210 mV only due to lower density of surface states. The work function of the kelvin probe was calibrated by a freshly cleaved highly ordered pyrolytic graphite surface, which has known work function of 4.65 eV (ref. 47).

We performed Kelvin probe measurements under dark and light condition (at different light intensities) to gauge the band bending near the surface of the perovskite film. We show a schematic band diagram for the generation of surface photovoltage at the perovskite surface in Fig. 4a. When the trap states are spatially located at the surface of a semiconductor thin film, accumulation of a particular type of charge can occur on the surface of the material. If the surface states are of acceptor type, then they are neutral when empty and will accumulate electrons; if they are donor type they will accumulate holes. This phenomenon gives rise to surface band bending due to the local depletion of the carriers from within the perovskite semiconductor, which become localized in the surface states (Fig. 4a). The higher the density of surface states is, the greater the depletion and the larger the magnitude of the band bending are. The direction of the band bending depends on the type of charge that is accumulated at the surface. The band bending which we illustrate in the Fig. 4a, occurs due to trapping of electrons in acceptor type surface states^38^ where negative charges accumulate on the surface leaving behind a positive space charge region close to the surface. Under illumination, the band-to-band (or trap to band) transition generates excess carriers. Due to the electric field created by the surface space charge region, photogenerated holes are swept towards the surface, which act to screen the trapped electron thus reducing the degree of band bending^38^,^39^. Under sufficient intensity of illumination, flat band condition can be achieved when the photogenerated hole density near the surface matches the density of trapped surface charge. We illustrate the situation under moderate illumination by the dotted lines in Fig. 4a. The difference in surface work-function (measured by a kelvin probe) in the dark and under illumination (known as surface photovoltage, SPV) represents change in the magnitude of the band bending at the surface of the semiconductor, and thus is indicative of the density of surface states. In Fig. 4b we show SPV measurements of the perovskite films. Both control perovskite and HPA treated films on FTO-coated glass show a continuous decrease in the work function as the light intensity is increased. The sign of the SPV signal suggests that the predominant surface states are acceptor type, which is manifested by a downward-bent band (from the perspective of the surface moving into the film) as we have illustrated in (Fig. 4a). We found that the difference on SPV between dark and illuminated of the control and HPA-processed films were 430 and 210 mV, respectively (Fig. 4b). The lower magnitude of SPV signal which we observe on the HPA-processed film suggests that HPA addition lowers the density of acceptor type trap states at the surface. We note here that a buried interface (FTO:perovskite herein) can also affect the SPV signal^38^ if selective transfer of one type of photogenerated carriers occurs at that heterojunction interface, or if there is a significant density of trap sites at the buried interface. However, we have shown in our previous work that in steady-state condition zero photocurrent is obtained for devices with FTO:perovskite interface, consistent with a non-selective nature of the interface^40^, unlike the compact TiO_2_:perovskite interface. In addition, we observe non-zero SPV signals at low light intensities (Fig. 4b) where the HPA-processed films have higher SPV value compared with the control films and the SPV signals do not change sign with higher intensities consistent with the SPV signal being dominated by the front surface. Thus the effect from the buried interface (if any) does not affect the conclusion we draw here.

**Figure 5:**
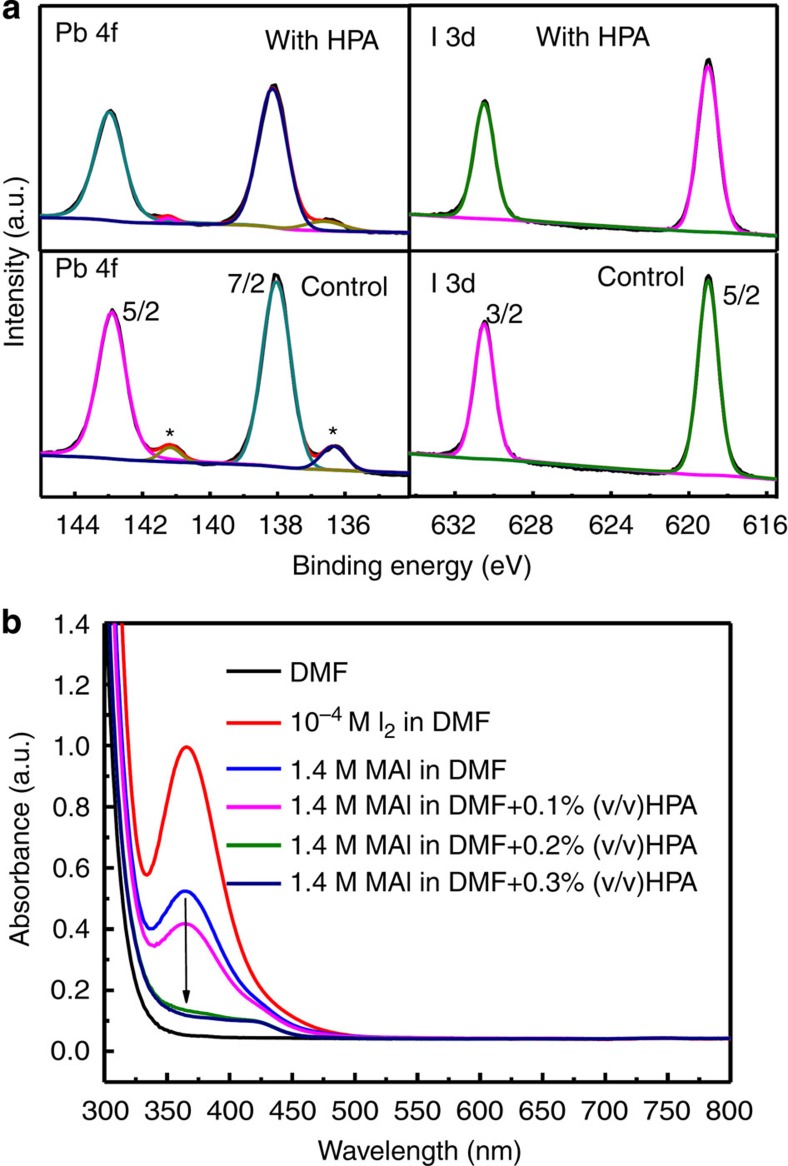
The possible origin of electronic trap states. (**a**) The high-resolution XPS spectra of Pb 4f and I 3d detail spectra for the perovskite films deposited on c-TiO_2_ coated FTO glass prepared from the precursor solution without (control) and with HPA. Peak pertaining to unsaturated Pb has been marked with star in the figure, which becomes smaller after addition of HPA in the precursor. (**b**) Ultraviolet–visible (UV–vis) absorption spectra of MAI or I_2_ dissolved in DMF and absorption quenching of MAI solution by adding HPA.

To elucidate the origin of these electronic trap states, we performed XPS analysis to map out the elemental composition of control and HPA-modified perovskite films, as we show in Fig. 5. We do not observe a signal for phosphorous in the film (Supplementary Fig. 3), indicating that HPA is not present above 0.1% concentration in the perovskite film after processing, notably with the detection limited to the surface region of the film. Hence, this implies that HPA does not physically participate in passivation of surface defects in the film. We note that from these measurements we cannot exclude the possible presence of phosphorous throughout the bulk of the film. From the XPS spectra of the signals for Pb 4f and I 3d, we estimate the I/Pb ratio in the film to be 2.19 and 2.50 for the control sample and sample with HPA, respectively. This stoichiometry indicates there is iodide deficiency in the surface region of both films (we expect a 3:1 ratio in the stoichiometric crystal). However, this deficiency is more exaggerated in the control film, implying that HPA partially restores the I/Pb stoichiometry in the perovskite film. We present another important observation in high-resolution spectra of binding energy pertaining to Pb in Fig. 5a. There are two main peaks in Pb 4f spectra: the Pb 4f_7/2_ and Pb 4f_5/2_ peaks^41^. We attribute the small peaks at around 136.3 and 141.2 eV to the presence of unsaturated Pb which has recently been assigned to metallic Pb (Pb^0^)^42^,^43^, marked star in Fig. 5a. This could simply be due to the fact that highly unsaturated Pb may show metallic characteristics. The presence of a substantial amount of unsaturated Pb is a further indication of the existence of iodide deficiencies in the perovskite lattice of the control sample, and the metallic lead species in the film are likely to act as recombination centres^44^. However, in the HPA-modified perovskite film, there seems to be a substantial reduction in the peak assigned to the unsaturated lead, and the peak area ratio of Pb^0^/Pb4f decreases from 10.9% (control) to 5.6% (with HPA). All of the experimental evidence presented thus far points towards there being much lower possibility of iodide vacancies in the in the crystal lattice of the HPA-processed films combined with a lower fraction of metallic lead in the films. It is very likely that further reduction of the metallic lead content is critical to realize perfect electronic quality of the perovskite films.

To further explore the possible origin of the iodide deficiency in perovskite film, which presumably leads to the generation of metallic lead, we studied the ultraviolet–visible absorption curve of the MAI solution in *N*,*N*-Dimethylformamide, using I_2_ solution as the standard reference. As we show in Fig. 5b, we observe a peak around 365 nm for the as mixed MAI solution, which can be attributed to the absorption of I_2_. We also clearly observe that the peak around 365 nm is decreased by adding HPA in the MAI solution. Using this absorption spectra we estimate the amount of I_2_ in MAI to be 5.26 × 10^−5^ M, based on measured calibration curve of I_2_ absorption at 365 nm (Supplementary Fig. 4). Thus, the ratio of I_2_ to MAI is only 37.5 parts per million atoms (p.p.m.a). It is quite impressive that such trace amount of impurity has such a large impact on the quality of the film. The role of HPA in promoting the film quality could thus be reducing the trace amount of I_2_ existing in MAI. We note, however, that on heating the pre-crystallized films, generation of I_2_ may be accelerated, and this may be the point at which the HPA is most critical.

### Photovoltaic performance and characterization

Finally, we fabricated planar heterojunction perovskite solar cells composed of FTO glass/c-TiO_2_/perovskite thin film/spiro-OMeTAD/Ag. In Supplementary Fig. 5 we show the dependence of the device performance with varying precursor concentration. We found that a 30 wt% precursor solution gives the best PCE, after which the device performance drops sharply with increasing concentration. We therefore fixed the precursor concentration at 30 wt%, and added HPA over a range of molar ratios of HPA to PbAc_2_·3H_2_O (Supplementary Table 1). We summarize the solar cell performance results, at optimized HPA concentration (molar ratio of 7.5% for HPA/PbAc_2_·3H_2_O), with s.d. in Table 1. We extracted the efficiencies quoted from measuring the current-voltage curve under simulated AM 1.5 sunlight at 100 mW cm^−2^ irradiance scanned from forward bias to short-circuit conditions.

Interestingly, we observed that the photocurrent is greatly enhanced by adding HPA into the precursor solution, which is consistent with the reduction of defect sites that could partake in trap-assisted recombination. At the optimal molar ratio of HPA/PbAc_2_·3H_2_O, the average short-circuit current density (*J*_*sc*_) reaches a value of 20.1 mA cm^−2^. This increase is impressive considering the film thickness is only 200 nm, which is much thinner than what is used in most of the highly efficient planar heterojuction devices, which show an optimum film thickness around 350–500 nm. The enhancement in *J*_*sc*_ is what leads to the improvement in the PCE, since the open-circuit voltage (*V*_*oc*_) and fill factor (FF) are only marginally improved. We obtain an average PCE of 15.6% at the optimized HPA concentration, which corresponds to an increase of ∼54% as compared with the control devices, which were fabricated without the addition of HPA. These devices have an average PCE of 10.1%. The champion cell shows excellent performance with a *J*_*sc*_ of 20.4 mA cm^−2^, a *V*_*oc*_ of 1.07 V and a FF of 0.74, leading to a PCE of 16.2%, greatly outperforming the champion control cell which has a *J*_*sc*_ of 17.2 mA cm^−2^, a *V*_*oc*_ of 1.07 V and a FF of 0.72, leading to a PCE of 13.2%. Also, the much narrower s.d., which we observe in the group with HPA suggests that the device performance is more reproducible as compared with the control devices without HPA. We note that in our previous work where we employed PbAc_2_·3H_2_O with lab-made MAI, we showed an average PCE of 14.0% with a champion cell PCE of 15.2% (ref. 27). However, as we explained near the start of the results section, for our previous work we employed MAI, which had not been purified via recrystallization. We expect that HPA, present as an impurity (residual from the HI solution), was responsible for ‘enhancing' the previously reported solar cells and they can therefore not be viewed as ‘HPA-free' device and compared directly with the control device, which we show in the present work. For the same reason, we emphasize again that the high-efficiency devices that we have previously reported fabricated employing the mixed halide lead precursor route (PbCl_2_+3 MAI) could have also been partially enhanced due to the contribution of HPA in the lab-made non-recrystallized MAI power which we have employed throughout all our previous work^9^,^10^,^45^, although neither at an optimal concentration nor a controlled level. This is supported by the results which we show below and in Supplementary Table 2.

It is a widely observed phenomenon that perovskite solar cells exhibit hysteresis in their current-voltage curves. As such, an important measurement by which devices can be compared, is to hold the cell at a fixed forward applied bias close to the maximum power point on the current-voltage curve, and to measure the power output over time until it reaches a steady stabilized value^46^. As we show in Table 1, the stabilized power output (SPO) is greatly enhanced with the addition of HPA, from an average SPO of 7.2% (control with no HPA) to 13.1% (with HPA). The champion cell with HPA gives a SPO of 14.1%, which is also much higher than the champion control device in the absence of HPA showing a SPO of 10.6% (see also in Supplementary Fig. 6).

To determine whether this ‘HPA effect' is ubiquitous, we added HPA to a precursor solution utilizing lead chloride as the lead salt. We observed a similar increase in both scanned and stabilized power conversion efficiencies (see Supplementary Table 2), which suggests that there is likely to be a universal effect of HPA on improving device performance, independent on thin-film deposition technique.

Before we conclude that the reducing character of HPA is the sole reason for retaining the I/Pb stoichiometry in the perovskite, we used non-reducing acids, such as hydrochloric acid (HCl) and acetic acid (HAc) or water (H_2_O; since HPA used in this work is a 50 wt% aqueous solution) as additives and we followed similar procedures to fabricate the perovskite films. We show the device performance in Supplementary Fig. 7. In contrast to the addition of HPA, the device performance drops sharply by addition of other additives in the precursor solution. This experiment is consistent with the function of HPA acting as a reducing agent, rather than as an acid, and also rules out the possible positive contribution of the water in HPA solution on the properties of the perovskite thin film and device performance.

Considering all the facts discussed so far, we attribute the reduction of the electronic defect density to the combined effect of reducing the non-stoichiometric defect sites, most particular a lower fraction of metallic lead, and the formation of larger grains. We attribute the latter to the slower crystallization rate (Supplementary Fig. 8), which we observe in perovskite films processed with the HPA additive.

## Discussion

We have demonstrated that the addition of hypophosphorous acid (HPA) to the perovskite precursor solution can greatly improve the quality of CH_3_NH_3_PbI_3_ perovskite thin films by reducing the energetic defects in the crystal and enhancing the polycrystalline grain morphology. This leads to the enhanced photoluminescence and solar cell device performance with improved reproducibility. A key observation is that the relative fraction of metallic lead in the perovskite films is greatly reduced via processing with HPA. This work identifies a specific crystallization enhancement additive, which improves the quality of the semiconductor, but it also highlights that much further scope remains to achieve entirely electronically homogeneous polycrystalline thin films. Understanding the subtleties of crystallization from the composition of the solutions to the final crystallized films is still in its infancy, and we expect that the discovery of other ‘impurity' additives to be important for the ongoing advancement of the perovskite technology. More directly, our work is likely to motivate other groups employing purified MAI to further boost their champion device performance beyond current world record efficiencies by adding HPA in their precursor solution.

## Methods

### Perovskite solution preparation

Methylamine iodide (MAI) was purchased from Dyesol and used as received. Lead acetate trihydrate (PbAc_2_·3H_2_O) (99.999% trace metals basis, CAS No. 6080-56-4) and hypophosphorous acid (HPA) solution (50 wt% in water, CAS No. 6303-21-5) were purchased from Sigma-Aldrich. To create the perovskite precursor solution, MAI and PbAc_2_·3H_2_O were dissolved in anhydrous *N*,*N*-Dimethylformamide at a 3:1 molar ratio with final concentrations of 28–40 wt%. Then HPA solution was added in the precursor solution with a molar ratio of HPA/PbAc_2_·3H_2_O in the range of 5.0–10.0%.

### Substrate preparation

Devices were fabricated on FTO-coated glass (Pilkington, 7Ω/square). Initially, FTO was removed from regions under the anode contact by etching the FTO with 2 M HCl and zinc powder. Substrates were then cleaned sequentially in 2% Hellmanex detergent, acetone, propan-2-ol and oxygen plasma. A hole-blocking layer of compact TiO_2_ was deposited by spin-coating a mildly acidic solution of titanium isopropoxide in ethanol, and annealed at 500 °C for 30 min. Spin-coating was carried out at 2,000 r.p.m for 45 s.

### Perovskite deposition

For devices, the perovskites were prepared by spin coating a perovskite solution at 2,000 r.p.m for 45 s in a nitrogen-filled glovebox. After spin-coating, the films were left on the bench drying for 10–15 min then annealed at 100 °C for 5 min. 7 wt% spiro-OMeTAD hole-transporting layer was then deposited from a chlorobenzene solution containing additives of lithium bis(trifluoromethanesulfonyl)imide and 4-tert-butylpyridine at 2,000 r.p.m for 45 s. Finally, 120-nm silver electrodes were thermally evaporated under vacuum of ∼10^−6^ torr, at a rate of ∼0.1 nm s^−1^, to complete the devices.

### X-ray diffraction

2*θ* scans were obtained from samples of perovskite deposited on the compact-TiO_2_ coated FTO glass using a Panalytical X'PERT Pro X-ray powder diffractometer with Cu *K*_*α1*_ (1.54060 Å).

### SEM

A field emission scanning electron microscope (SEM; Hitachi S-4300) was used to acquire SEM images. The instrument uses an electron beam accelerated at 2.0 kV, enabling operation at a variety of currents.

### Surface photovoltage and Kelvin probe

Vibrating Kelvin Probe (probe diameter=2 mm) (KP Technology, UK) was used to determine the surface potential. Samples were prepared by depositing perovskite films (thickness around 200 nm) on FTO glasses. SPV measurements in ambient were done under illumination from a 150-W quartz tungsten halogen lamp, where the intensity was varied linearly by controlling the power supplied to the lamp. Using an optical fibre and lens, light beam fall on the perovskite surface at an angle of 45° (with respect to the sample surface) to illuminate the whole surface area. This configuration allowed us to do the illumination and probing from the same side, that is, perovskite:air interface side. For each category, the measurements were done on different spots and different samples and the average value is reported. The work function of the kelvin probe was calibrated by a freshly cleaved highly ordered pyrolytic graphite surface.

### Ultraviolet–visible

The absorbance of the perovskite films on glass were measured on a Carry 300 Bio (Agilent Technologies). To reduce the sample variance, at least three samples were determined for each group and the average of all spectra presented.

### Photothermal deflection spectroscopy

For this particular measurement, we made use of quartz rather than the FTO-coated glass to minimize light absorption due to the substrate. During the measurement we kept the samples in a hermetically sealed quartz cuvette filled with an inert liquid, Fluorinert FC-72 from 3M Corporation, which acts as deflection medium with high-temperature-dependent refractive index. We excited the perovskite films from the quartz side with a modulated monochromated light beam perpendicular to the plane of the sample. Modulated monochromated light beam was produced by a combination of a Light Support MKII 100 W Xenon arc source and a CVI DK240 monochromator. The transverse probe beam was produced with Qioptiq 670-nm fibre-coupled diode laser and passed as close as possible to the perovskite-film surface. Beam deflection was measured using a differentially amplified quadrant photodiode and a Stanford Research SR830 lock-in amplifier.

### X-ray photoemission spectroscopy

x-ray photoelectron spectroscopy measurements were conducted using the PHI 5300ESCA Perkin-Elmer spectrometer. The spectra were calibrated with the C1s peak (284.66 eV).

### Photoluminescence

Samples consisted of perovskite films prepared on glass. Steady-state and time-resolved PL measurements were performed using a time-resolved single-photon counting setup (FluoTime 300, PicoQuant GmbH). Samples were photoexcited using a 507-nm laser head (LDH-P-C-510, PicoQuant GmbH) at frequencies between 0.2–10 MHz with a pulse duration of 117 ps and fluence of ∼0.3 μJ cm^−2^.

### Photoluminescence quantum efficiency

PLQE measurements were taken in an integrating sphere using established techniques. The samples were photoexcited using a 532-nm CW laser operating at a fluence of 200 mW cm^−2^ (equivalent to ∼3 suns).

### Fluorescence microscopy

Optical microscopy and spectroscopy were performed using a custom sample scanning confocal microscope built around a Nikon TE-2000 inverted microscope fitted with an infinity corrected 50 × dry objective (Nikon L Plan, NA 0.7, CC 0–1.2). We measured the point spread function of our systems to be 348 nm FWHM. A 470-nm pulsed diode laser (PDL-800 LDH-P-C-470B, 300 ps pulse width), fluence of∼1 μJ cm^−2^ per pulse was used for excitation with repetition rate of 500 kHz for time-resolved PL measurements and 40 MHz for collecting fluorescence images. The emission was filtered through a 50/50 dichroic beamsplitter and a 700–850 nm bandpass filter (700 LP and 850 SP). PL from the sample was directed to a Micro Photon Devices (MPD) PDM Series single photon avalanche photodiode with a 50-μm active area. The sample stage was controlled using a piezo controller (Physik Instrumente E-710). For fluorescence images, the pixel step size was set to 100 nm with a pixel dwell time (integration time) of 100 ms.

### Solar cell characterization

The current density−voltage (*J−V*) curves were measured (2400 Series SourceMeter, Keithley Instruments) under simulated AM 1.5 sunlight at 100 mW cm^−2^ irradiance generated by an Abet Class AAB sun 2000 simulator, with the intensity calibrated with an NREL calibrated KG5 filtered Si reference cell. The mismatch factor was calculated to be <1%. The solar cells were masked with a metal aperture to define the active area, typically 0.0919, cm^2^ (measured individually for each mask) and measured in a light-tight sample holder to minimize any edge effects and ensure that the reference cell and test cell were located in the same spot under the solar simulator during measurement. The ‘stabilized power output' of the devices versus time was measured under load near the maximum power point at the same conditions.

## Additional information

**How to cite this article**: Zhang, W. *et al*. Enhanced optoelectronic quality of perovskite thin films with hypophosphorous acid for planar heterojunction solar cells. *Nat. Commun.* 6:10030 doi: 10.1038/ncomms10030 (2015).

## Supplementary Material

Supplementary Information Supplementary Figures 1-8, Supplementary Tables 1-2 and Supplementary Note 1.

## Figures and Tables

**Table 1:** Comparison of solar cell performance parameters without (control) and with HPA.

Sample	*J*_*sc*_ (mA cm^−2^)	PCE (%)	*V*_*oc*_ (V)	FF	SPO (%)
Control					
Average	14.5±1.7	10.1±2.2	1.01±0.07	0.67±0.05	7.2±2.9
Champion	17.2	13.2	1.07	0.72	10.6
with HPA					
Average	20.1±0.4	15.6±0.4	1.07±0.02	0.72±0.02	13.1±0.5
Champion	20.4	16.2	1.07	0.74	14.1

FF, fill factor; *J*_sc_, short-circuit current density; PCE, power conversion efficiency; SPO, stabilized power output; *V*_oc_, open-circuit voltage

Average photovoltaic parameters with s.d. were obtained based on 16 cells for each set.
