# Supplementary material for: Enhanced optoelectronic quality of perovskite thin films with hypophosphorous acid for planar heterojunction solar cells
Source: Nat Commun. 2015 Nov 30;6:10030. doi: 10.1038/ncomms10030 (PMC4674686; doi:10.1038/ncomms10030)
Supplement: Supplementary Information — Supplementary Figures 1-8, Supplementary Tables 1-2 and Supplementary Note 1. [file ncomms10030-s1.pdf]

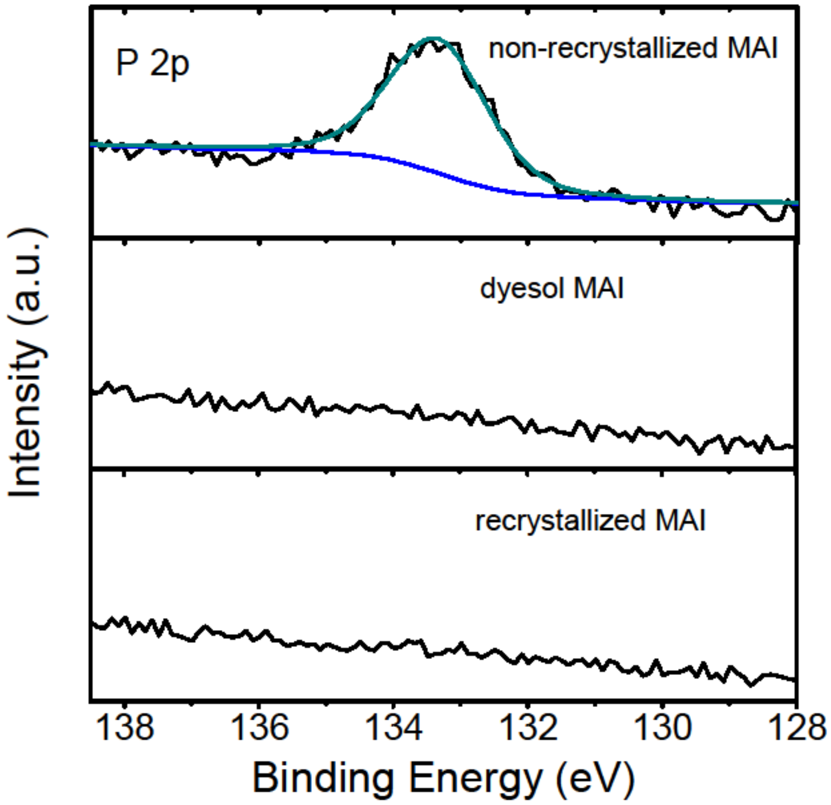

Supplementary Figure 1. XPS analysis for MAI powders. The high-resolution XPS spectra of P 2p detail spectra for the MAI powders prepared in the lab without/with recrystallization or purchased from dyesol company.

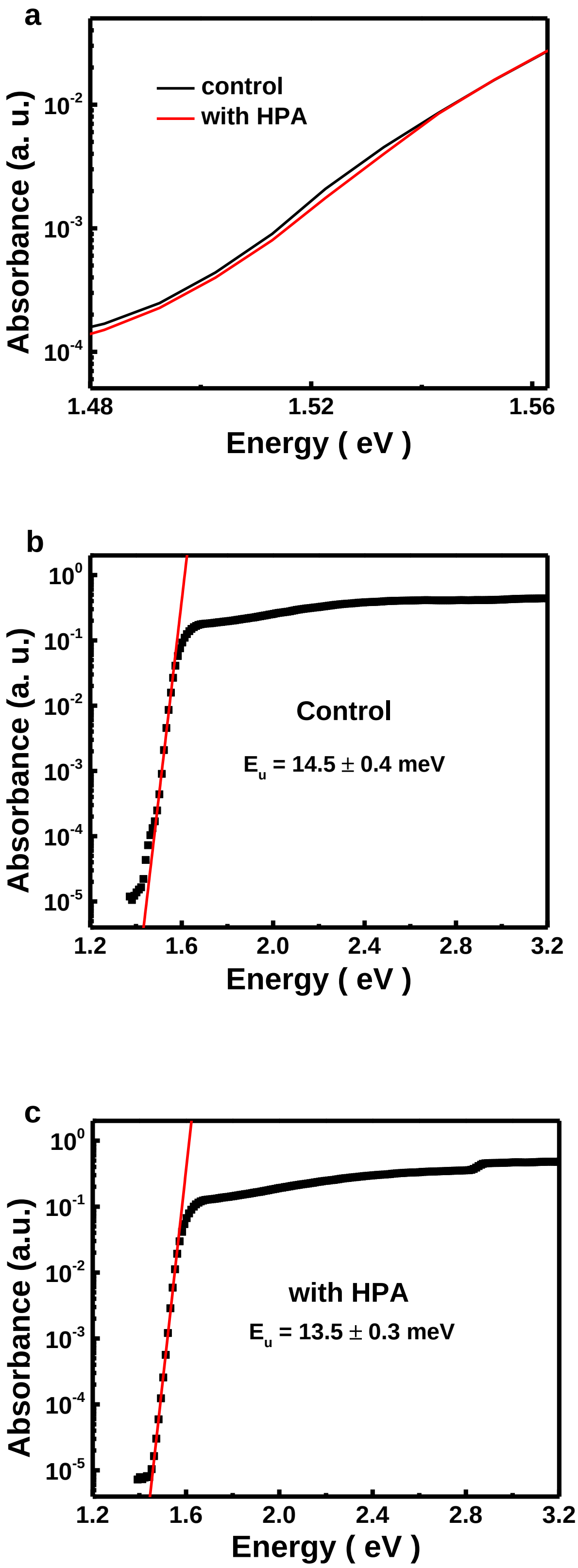

Supplementary Figure 2. Urbach energy analysis. (a) PDS spectra of the perovskite film processed without and with HPA deposited on quartz substrate. Linear fits used to calculate the Urbach energy 'Eu' for the perovskite film processed without (b) and with HPA (c).

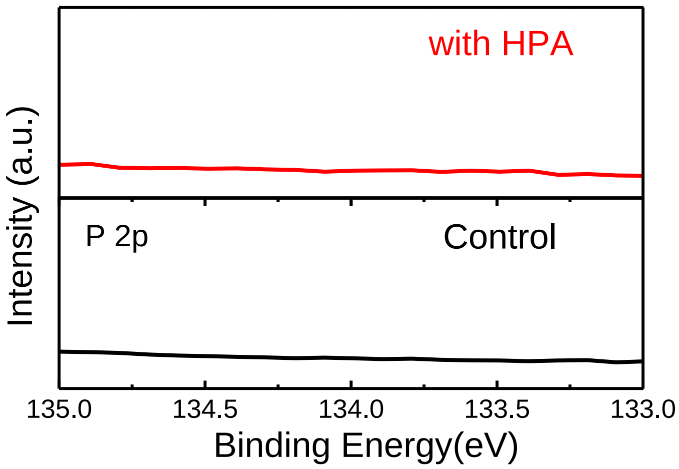

Supplementary Figure 3. XPS analysis for the perovskite thin films. The high-resolution XPS spectra of P 2p detail spectra for the perovskite films deposited on c-TiO_2 coated FTO glass prepared from the precursor solution without (control) and with HPA.

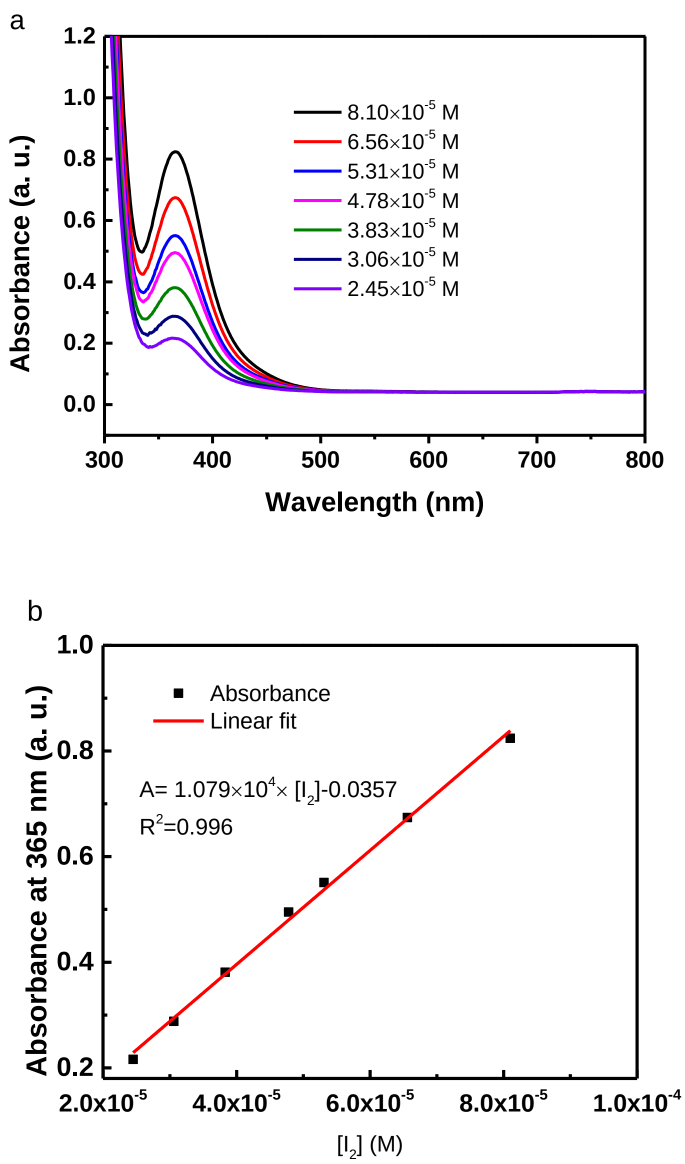

Supplementary Figure 4. Estimation of I_2 concentration. UV-Vis absorption spectra of I_2 dissolved in DMF at different concentrations (a) and the corresponding calibration curve based on the absorption at 365 nm.

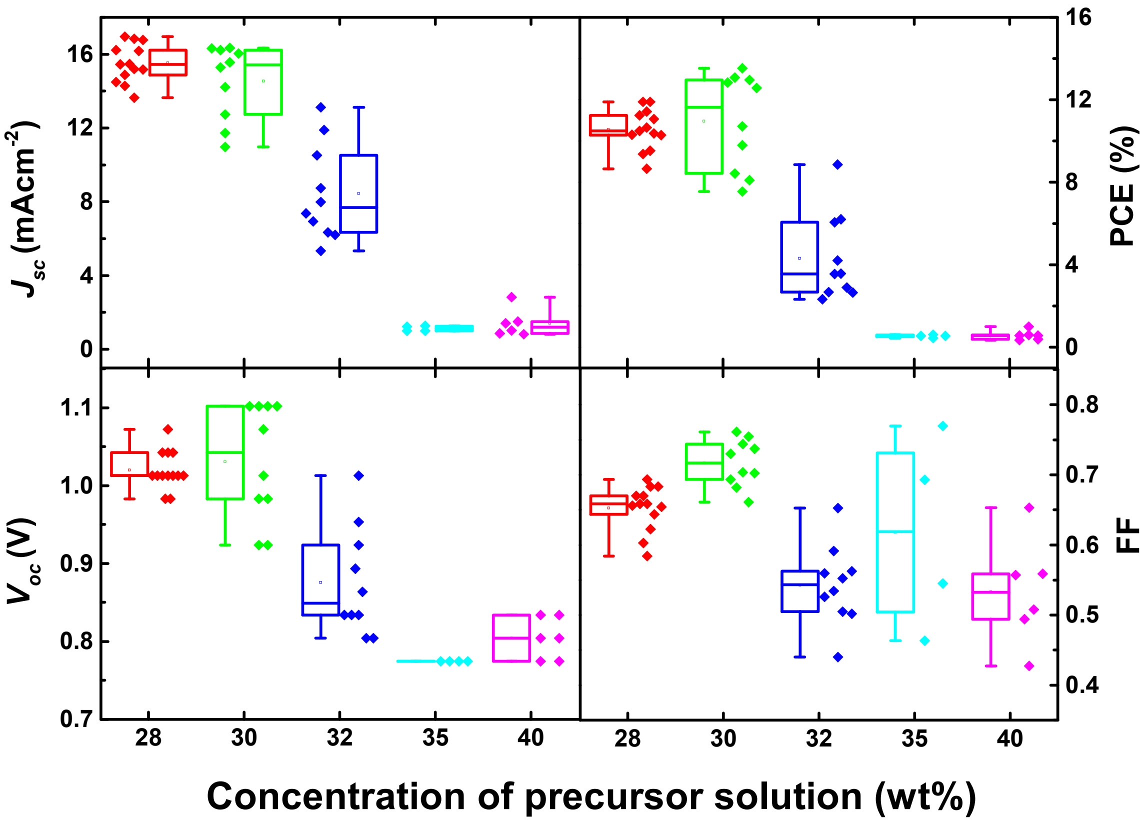

Supplementary Figure 5. Photovoltaic performance of solar cells. Dependence of device performance on the concentration of perovskite precursor solution. The performance parameters are extracted from forward bias to short-circuit current voltage curves of perovskite solar cells measured under simulated AM1.5 sun light at 100 mW cm^{-2}. The data are represented as a standard box plot where the box range is defined by the standard deviation (s.d.). Ninety percent of all data points fall within the upper and lower whisker.

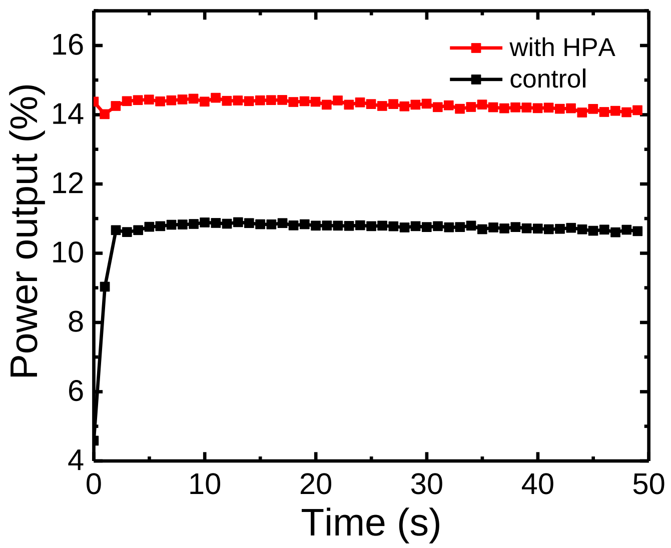

Supplementary Figure 6. Photovoltaic performance of champion cells. Power output over time of the champion cells processed without (control) and with HPA, held under constant applied forward bias close to the maximum power point voltage on the current voltage curve.

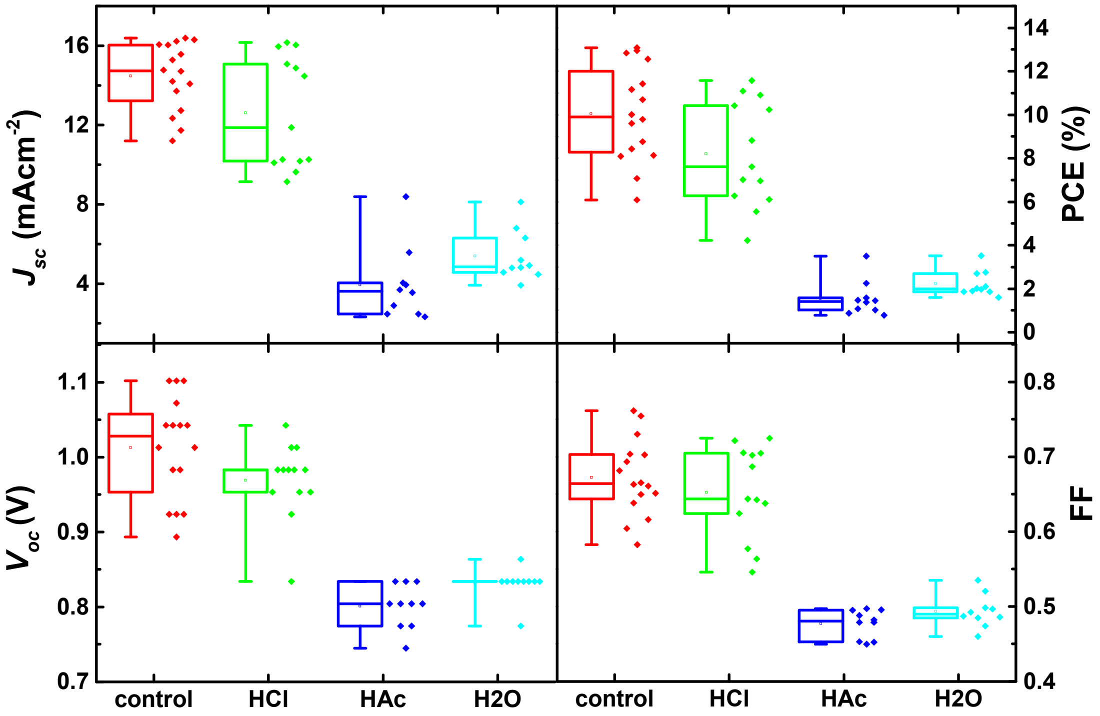

Supplementary Figure 7. Effect of other additives. The influence of hydrochloric aicd (HCl), acetic acid (HAc) and water (H_2O) upon the performance of perovskite solar cells. The performance parameters are extracted from forward bias to short-circuit current voltage curves of perovskite solar cells measured under simulated AM1.5 sun light at 100 mW cm^{-2}. The data are represented as a standard box plot where the box range is defined by the standard deviation (s.d.). Ninety percent of all data points fall within the upper and lower whisker.

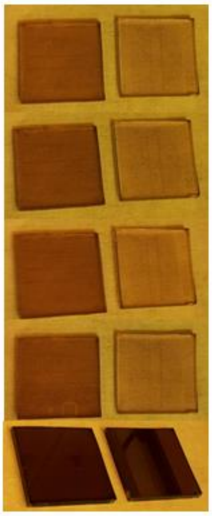

Supplementary Figure 8. Effect of drying on crystallization. Photographs of as spin-coated perovskite films without (control) and with addition of HPA with varied drying time in glovebox and mirror-like films after annealing at 100 degree for 5 min in glovebox.

Supplementary Table1 | Solar cell performance parameters for different HPA concentrations. Average photovoltaic parameters with standard deviations were obtained based on 16 cells for each set.

Molar ratio of HPA/$PbAc_2$ (%)	J_{sc} (mA cm^{-2}) [a]	PCE (%)[b]	V_{oc} (V) [c]	FF [d]
0.0	14.5 ± 1.7	10.1 ± 2.2	1.01 ± 0.07	0.67 ± 0.05
5.0	17.4 ± 1.1	12.9 ± 1.4	1.05 ± 0.02	0.69 ± 0.03
6.25	18.8 ± 1.3	13.6 ± 1.0	1.05 ± 0.02	0.68 ± 0.02
7.5	20.0 ± 0.5	15.6 ± 0.4	1.07 ± 0.02	0.72 ± 0.02
8.75	18.1 ± 1.0	13.1 ± 1.0	1.03 ± 0.03	0.70 ± 0.03
10.0	17.1 ± 1.3	11.0 ± 1.2	1.01 ± 0.04	0.63 ± 0.04

[a] J_{sc}, short-circuit current density; [b] PCE, power conversion efficiency; [c] V_{oc}, open circuit voltage; [d] FF, fill factor.

Supplementary Table 2 | Solar cell performance parameters without (control) and with HPA using $PbCl_2$ as the lead salt. Average photovoltaic parameters with standard deviations were obtained based on 16 cells for each set.

sample		J_{sc} (mA cm^{-2}) [a]	PCE (%)[b]	V_{oc} (V) [c]	FF [d]	SPO (%)[e]
control	average	19.1±0.6	10.1±1.2	0.98±0.04	0.54± 0.07	8.2±1.5
	champion	18.6	11.5	1.01	0.61	9.9
with HPA	average	19.7 ± 1.0	13.8±0.76	1.05± 0.02	0.67± 0.02	12.8±0.4
	champion	21.0	15.0	1.07	0.68	13.0

[a] J_{sc}, short-circuit current density; [b] PCE, power conversion efficiency; [c] V_{oc}, open circuit voltage; [d] FF, fill factor; [e] SPO, stabilized power output.

Supplementary Note 1

Theoretical model for description of photo-luminescence decay

The model described in this section is in many respects similar to the one described in [37]. We reformulate the model to make it more suitable for the case discussed in the current study. The main goal of the modelling is to estimate the concentration of electronic traps by analysing the PL intensity decay in the perovskite material. The electronic traps most likely are associated with atomic vacancies producing electronic levels deep in the band gap. Such levels usually serve as rather efficient electron-hole recombination (Shockley-Read-Hall) centres and are referred below as to 'traps'. Many experimental studies give the evidence for a mechanism of deactivation of these recombination centres in perovskite material, possibly due to formation of the long-living vacancy-electron complexes, which we call 'filled traps'. As all the PL decay measurements were carried out at the steady state (long after all slow transients have relaxed), we may take both the total and filled trap concentrations in the material to be constant during the PL measurement cycle. In this case we focus the analysis on the kinetics of charge carrier and exciton concentrations only.

Within this framework we can then write a set of equations for time evolution of free electrons N_e and excitons N_x

$$\frac{dN_e}{dt} = r_d N_x - r_f N_e \cdot (N_e + n_T) - r_{eh} \cdot N_e \cdot (N_e + n_T) \tag{1a}$$

$$\frac{dN_x}{dt} = r_f N_e \cdot (N_e + n_T) - r_d N_x - r_x N_x \tag{1b}$$

where r_f and r_d are exciton formation and dissociation rates, respectively, r_{eh} is the total electron-hole recombination rate, which includes both direct band-to-band recombination and the one associated with empty electronic traps (Shockley-Read-Hall centers), r_x is the decay rate of excitons (radiative or non-radiative), and n_T is the filled trap concentration. Eqs (1) can be simplified using the concept of thermal equilibrium between charge carriers and excitons (for details see Supplemental Materials in [37])

$$N_e \cdot N_h - B \cdot N_x = 0,\ B = \frac{v_x}{v_h v_e} \exp\left[-\frac{E_x}{k_B T}\right] \tag{2}$$

where E_x is the exciton binding energy. Using the notations $N = N_e + N_x$ and $N_h = N_e + n_T$ this equation can be rewritten as

$$N = \frac{1}{B}\cdot\left(N_{\mathrm{e}}^{2} + N_{\mathrm{e}}\cdot\left(B + n_{\mathrm{T}}\right)\right) \tag{3}$$

which allows expressing electronic concentration in terms of *N* as

$$N_{\mathrm{e}} = -\frac{\left(B + n_{\mathrm{T}}\right)}{2} + \frac{1}{2}\sqrt{\left(B + n_{\mathrm{T}}\right)^{2} + 4BN} \approx \frac{BN}{B + n_{\mathrm{T}}} \tag{4}$$

here we used an approximation $4BN/\left(B+n_{\mathrm{T}}\right)^{2} << 1$ taking into account that at $B \approx 4\cdot 10^{17}\ \mathrm{cm}^{-3}$ if $E_{\mathrm{x}} \approx 10\ \mathrm{meV}$ and $N(t) \leq N(t=0) \approx 10^{16}\ \mathrm{cm}^{-3}$. Then adding two Eqs (1) together and using Eqs (3) and (4) obtain

$$\frac{dN}{dt} = -\left(r_{\mathrm{eh}} + r_{\mathrm{x}}/B\right)\cdot\frac{BN}{B+n_{\mathrm{T}}}\cdot\left(\frac{BN}{B+n_{\mathrm{T}}} + n_{\mathrm{T}}\right) \tag{5}$$

This can be rewritten in terms of N_{e}

$$\frac{dN_{\mathrm{e}}}{dt} = -\gamma\cdot N_{\mathrm{e}}\cdot\left(N_{\mathrm{e}} + n_{\mathrm{T}}\right),\ \gamma = \left(r_{\mathrm{eh}} + r_{\mathrm{x}}/B\right)\cdot\frac{B}{B+n_{\mathrm{T}}} \tag{6}$$

Solution to this equation is

$$N_{\mathrm{e}} = \frac{n_{\mathrm{T}}\cdot N_{\mathrm{e}}(0)\cdot\exp\left(-\gamma n_{\mathrm{T}}\cdot t\right)}{n_{\mathrm{T}} + N_{\mathrm{e}}(0)\cdot\left(1-\exp\left(-\gamma n_{\mathrm{T}}\cdot t\right)\right)},\ N_{\mathrm{e}}(0) = N_{\mathrm{e}}(t=0) \tag{7}$$

Normalized time dependent PL intensity is given by

$$I_{\mathrm{PL}}(t) = \frac{N_{\mathrm{e}}\cdot N_{\mathrm{h}}}{N_{\mathrm{e}}(0)\cdot N_{\mathrm{h}}(0)} = \frac{n_{\mathrm{T}}^{2}\cdot\exp\left(-\gamma n_{\mathrm{T}}\cdot t\right)}{\left(n_{\mathrm{T}} + N_{\mathrm{e}}(0)\cdot\left(1-\exp\left(-\gamma n_{\mathrm{T}}\cdot t\right)\right)\right)^{2}} \tag{8}$$

For the fitting procedure it is convenient to use the expression

$$I_{\mathrm{PL}}(t) = \alpha\cdot\frac{\exp\left(-\gamma n_{\mathrm{T}}\cdot t\right)}{\left(1 + \frac{N_{\mathrm{e}}(0)}{n_{\mathrm{T}}}\cdot\left(1-\exp\left(-\gamma n_{\mathrm{T}}\cdot t\right)\right)\right)^{2}} + \beta \tag{9}$$

where we introduced extra two parameters α, and β. The parameter α is introduced to account for the fast PL decay at times shorter than 8 ns (see Fig. 2d), which origin is unknown. The other parameter, β, takes into account the noise level of the instrument. The parameters of our interest are $N_{\mathrm{e}}(0)/n_{\mathrm{T}}$ and $\gamma\cdot n_{\mathrm{T}}$, which values are given in the caption to Fig. 2d. It can be shown that Eq. (9) describes both the extreme cases of mono-molecular ($N_{\mathrm{e}}(0) << n_{\mathrm{T}}$) and bimolecular ($N_{\mathrm{e}}(0) >> n_{\mathrm{T}}$ and $\gamma n_{\mathrm{T}}\cdot t_{0} << 1$) recombination and all the intermediate regimes.

According to Fig. 2d the function given by Eq. (9) approximates very well the experimental curves. In particular, for the control sample (black curve in Fig. 2d) fitting with Eq.

(9) (grey line) allowed obtaining the parameter values $N_{\mathrm{e}}(0)/n_{\mathrm{T}}=0.634$ giving together with $N_{\mathrm{e}}(0)=N(0)=10^{16}\,\mathrm{cm}^{-3}$ the value $n_{\mathrm{T}}=1.58\cdot10^{16}\ \mathrm{cm}^{-3}$. Using the other fit parameter $\gamma\cdot n_{\mathrm{T}}=9.57\cdot10^{6}\ \mathrm{s}^{-1}$ we obtain $\gamma=6.0\cdot10^{-10}\ \mathrm{cm}^{3}\mathrm{s}^{-1}$. Note that the obtained value $n_{\mathrm{T}}<<B=4\cdot10^{17}\ \mathrm{cm}^{-3}$ validates our assumption about $N_{\mathrm{e}}(0)=N(0)$ (see Eq. (4)). By fitting the PL decay for the HPA treated sample (red curve in Fig. 2d) we found $N_{\mathrm{e}}(0)/n_{\mathrm{T}}=3.07$ and $\gamma\cdot n_{\mathrm{T}}=0.3\cdot10^{6}\ \mathrm{s}^{-1}$ giving the values $n_{\mathrm{T}}=0.325\cdot10^{16}\ \mathrm{cm}^{-3}$ and $\gamma=0.92\cdot10^{-10}\ \mathrm{cm}^{3}\mathrm{s}^{-1}$. These results indicate that the trap concentration drops about 5 times for the films processed with HPA. Simultaneously $\gamma=\left(r_{\mathrm{eh}}+r_{\mathrm{x}}/B\right)\cdot\dfrac{B}{B+n_{\mathrm{T}}}\approx\left(r_{\mathrm{eh}}+r_{\mathrm{x}}/B\right)$ also shows 6.5 times decrease most likely due to the decrease in r_{eh}, which may significantly exceed the exciton decay rate r_{x}/B, and has a significant contribution from empty trap-mediated (Shockley-Rheed-Hall) recombination proportional to $N_{\mathrm{T}}-n_{\mathrm{T}}$, where N_{T} is the total trap concentration. As compared to the control sample, the PL decay rate γ for the film processed with HPA should decrease by the factor roughly the same as the decrease in $N_{\mathrm{T}}-n_{\mathrm{T}}$, i.e. 5 times if we assume that n_{T} is proportional to N_{T}. This is consistent with the observed 6.5 times decrease in γ.

It is important to estimate an impact of the five-time decrease in n_{T} and γ on the PV device performance. If we compare the control and HPA treated devices at the same applied voltage then the difference in the device performance can entirely be due to the change in the current. The latter can be taken as proportional to the charge density. Therefore to estimate an impact of the trap density change it is enough to estimate the change in the photo-generated electron density. The latter can be estimated from the modified Eq. (6)

$$\frac{dN_{\mathrm{e}}}{dt}=J-\gamma\cdot N_{\mathrm{e}}\cdot\left(N_{\mathrm{e}}+n_{\mathrm{T}}\right) \tag{10}$$

where J is the electron photo-generation rate (number density of the absorbed light). Using this equation in the steady state regime obtain the expression for the electron density

$$J-\gamma\cdot N_{\mathrm{e}}\cdot\left(N_{\mathrm{e}}+n_{\mathrm{T}}\right)=0\rightarrow N_{\mathrm{e}}=-\frac{n_{\mathrm{T}}}{2}+\sqrt{\left(\frac{n_{\mathrm{T}}}{2}\right)^{2}+\frac{J}{\gamma}} \tag{11}$$

Substitute here the values of n_{T} and γ for the control and HPA treated samples obtain

$$N_{\mathrm{e}}=7.26\cdot10^{15}\,\mathrm{cm}^{-3}\ \ \text{control} \tag{12a}$$

$$N_{\mathrm{e}}=7.37\cdot10^{16}\,\mathrm{cm}^{-3}\ \ \text{HPA-treated} \tag{12b}$$

which shows about an order of magnitude increase in electronic density for 200 nm thick sample under 1 Sun illumination ($J = 7.2 \cdot 10^{21}\ \mathrm{cm^{-3}s^{-1}}$). The described situation corresponds to open circuit conditions in real PV device. In the case of any closed external circuit the difference in the charge densities is going to be smaller due to the photo-current. However, by any means the increase in the charge density due to the decreased trap concentration upon HPA treatment should result in an increase in the PV device performance.
